# A 72-channel receive array coil allows whole-heart cine MRI in two breath holds

**DOI:** 10.1186/s41747-022-00305-w

**Published:** 2022-11-01

**Authors:** Hugo Klarenberg, Mark Gosselink, Bram F. Coolen, Tim Leiner, Aart J. Nederveen, Adrianus J. Bakermans, Hildo J. Lamb, S. Matthijs Boekholdt, Martijn Froeling, Gustav J. Strijkers

**Affiliations:** 1 Department of Biomedical Engineering and Physics, Amsterdam University Medical Centers, Amsterdam Cardiovascular Sciences, University of Amsterdam, Amsterdam, The Netherlands; 2 Department of Radiology, University Medical Center Utrecht, Utrecht, The Netherlands; 3 Department of Radiology and Nuclear Medicine, Amsterdam University Medical Centers, University of Amsterdam, Amsterdam, The Netherlands; 4 Department of Radiology, Leiden University Medical Center, Leiden, The Netherlands; 5 Department of Cardiology, Amsterdam University Medical Centers, Amsterdam Cardiovascular Sciences, University of Amsterdam, Amsterdam, The Netherlands

**Keywords:** Breath holding, Healthy volunteers, Heart ventricles, Magnetic resonance imaging (cine), Stroke volume

## Abstract

**Background:**

A new 72-channel receive array coil and sensitivity encoding, compressed (C-SENSE) and noncompressed (SENSE), were investigated to decrease the number of breath-holds (BHs) for cardiac magnetic resonance (CMR).

**Methods:**

Three-T CMRs were performed using the 72-channel coil with SENSE-2/4/6 and C-SENSE-2/4/6 accelerated short-axis cine two-dimensional balanced steady-state free precession sequences. A 16-channel coil with SENSE-2 served as reference. Ten healthy subjects were included. BH-time was kept under 15 s. Data were compared in terms of image quality, biventricular function, number of BHs, and scan times.

**Results:**

BHs decreased from 7 with C-SENSE-2 (scan time 70 s, 2 slices/BH) to 3 with C-SENSE-4 (scan time 42 s, 4–5 slices/BH) and 2 with C-SENSE-6 (scan time 28 s, 7 slices/BH). Compared to reference, image sharpness was similar for SENSE-2/4/6, slightly inferior for C-SENSE-2/4/6. Blood-to-myocardium contrast was unaffected. C-SENSE-4/6 was given lower qualitative median scores, but images were considered diagnostically adequate to excellent, with C-SENSE-6 suboptimal. Biventricular end-diastolic (EDV), end-systolic (ESV) and stroke volumes, ejection fractions (EF), cardiac outputs, and left ventricle (LV)-mass were similar for SENSE-2/4/6 with no systematic bias and clinically appropriate limits of agreements. C-SENSE slightly underestimated LV-EDV (-6.38 ± 6.0 mL, *p* < 0.047), LV-ESV (-7.94 ± 6.0 mL, *p* < 0.030) and overestimated LV-EF (3.16 ± 3.10%; *p* < 0.047) with C-SENSE-4. Bland-Altman analyses revealed minor systematic biases in these variables with C-SENSE-2/4/6 and for LV-mass with C-SENSE-6.

**Conclusions:**

Using the 72-channel coil, short-axis CMR for quantifying biventricular function was feasible in two BHs where SENSE slightly outperformed C-SENSE.

**Supplementary Information:**

The online version contains supplementary material available at 10.1186/s41747-022-00305-w.

## Key points

- The 72-channel coil permits quantification of cardiac function in two breath-holds.
- Functional parameters showed no mean bias and acceptable limits of agreement.
- Expert scoring deemed the faster imaging protocol of diagnostic quality.

## Background

Cardiac magnetic resonance (CMR) with cinematographic, in short “cine,” sequences is considered the reference standard for the quantitative assessment of left ventricle (LV) and right ventricle (RV) volumes, function, mass, as well as morphology [1]. Quantification of biventricular function and morphology is essential for the diagnosis and management of various heart diseases [2–5]. In routine practice, a two-dimensional CMR cine protocol, including survey scans, three long-axis slices (2-chamber view, 3-chamber view, and 4-chamber view), and approximately 14 contiguous short-axis slices covering the whole heart from base to apex, takes approximately 20 min.

Preferably, a retrospectively cardiac gated two-dimensional segmented k-space cine balanced steady-state free precession (bSSFP) sequence is used [6]. The advantage of bSSFP is that it provides superior blood pool to myocardium contrast with a high signal-to-noise ratio facilitating precise delineations of the left and right ventricular endo- and epicardial borders, wall trabeculation, and papillary muscles [7–9], which are required for quantitative evaluation of the myocardium. A downside of a bSSFP sequence, particularly at 3 T, is that images are susceptible to artifacts from rapid through-plane blood flow, banding artifacts from off-resonance effects, and artifacts due to respiratory motion [10]. To achieve sufficient spatiotemporal resolution and whole-heart coverage, the acquisition of a whole-heart short-axis cine acquisition is segmented over multiple (typically 7–9) breath holds (BHs) in typically 10–12 s. Planning and acquisition of cine images in other orientations require additional BHs.

Holding one’s breath can be difficult for some patients (*e.g.,* those suffering from heart failure), and respiratory motion-related artifacts due to noncompliance and incomplete BHs are common. A reduction in the number of BH required for a routine functional exam would increase patient comfort, reduce the loss of data due to motion artifacts, and save money by reducing scan time. Various acceleration strategies can be exploited to reduce the number of required k-lines and thus reducing the number of required BHs, while preserving spatiotemporal resolution. These include classical parallel imaging techniques in the image space, such as sensitivity encoding (SENSE) and array coil spatial sensitivity encoding, ASSET, or in k-space such as generalised autocalibrating partially parallel acquisition, GRAPPA, and autocalibrating reconstruction for Cartesian imaging, ARC [11, 12]. These techniques exploit sparsity in images like compressed sensing [13] and combinations thereof such as compressed-SENSE (C-SENSE) [14–18]. All these techniques will benefit from parallel imaging with a high number of receiver coils [19–22].

In this study, we aimed to reduce the imaging time of cine CMR using a recently developed 72-channel cardiac receive array coil in combination with standard vendor-supplied SENSE and C-SENSE acceleration sequences. We used C-SENSE acceleration factors of 2, 4, and 6 to reduce the number of BHs from 7 to as low as 3 and 2 for a whole-heart cine short-axis stack and validated biventricular function and morphology quantification with fewer BHs. Results were qualitatively and quantitatively compared with a standard protocol with a 16-channel receive array coil and SENSE with acceleration factor 2.

## Methods

### Subjects

Ten healthy subjects (five females), aged 31.8 ± 8.1 years (mean ± standard deviation), body weight 74.4 ± 9.9 kg, and heart rate 60.1 ± 7.3 beats/min were prospectively included. All experiments in this study adhered to the guidelines of the local Medical Ethical Committee and were performed in accordance with the declaration of Helsinki. All subjects provided written consent for participation prior to inclusion.

### Dedicated 72-channel receiver array coil

**Fig. 1:**
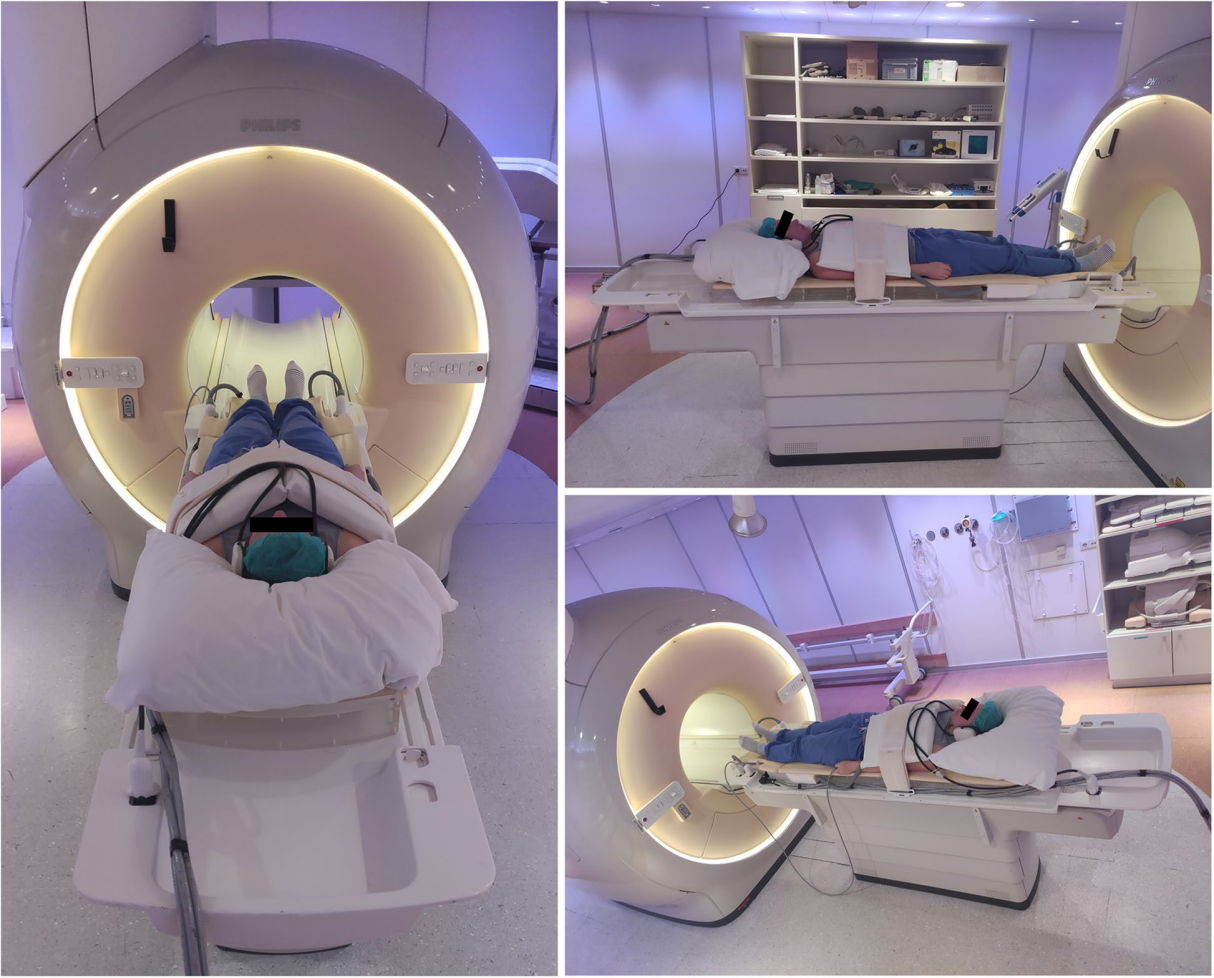
Photograph of a subject on the scanner bed with the 72-channel cardiac receive array coil placed on the chest

Figure 1 shows the setup of the dedicated 72-channel receive array coil [23]. The high-density interface box contains the preamplifiers which are placed directly on top of the digital receivers. All 72-receiver elements are connected to the pre-amplifiers via cable traps and pre-amplifier decoupling circuits. Although the coil loops are small, their sensitivity is sufficient to fully cover the thorax while maximising the signal-to-noise ratio and minimising the coil geometry factor (g-factor). The coil operates in conjunction with the standard vendor-supplied 12-channel posterior receive array coil integrated in the patient table, amounting to 84 receive channels in total.

### Data acquisition

All examinations were performed with a 3-T scanner (Ingenia, Philips Healthcare, Best, The Netherlands). After standard survey scans to plan short-axis and long-axis views, whole-heart short-axis cine images using an electrocardiographically gated bSSFP sequence were acquired, using several protocols detailed below.

First, a stack of 14 contiguous cine short-axis slices was acquired with the vendor-supplied 16-channel anterior receive array coil, in combination with the 12 channels posterior receiver array coil, and SENSE acceleration factor 2 requiring 7 BHs. BHs were carried out at mid-expiration after full inspiration. Subsequently, the subjects were repositioned back on the bed in the scanner with the 72-channel receiver array coil. After repeating the survey scans and replanning of all views, the stack of 14 cine short-axis slices were acquired with SENSE acceleration factors of 4 (3 BHs), 6 (2 BHs) as well as with C-SENSE-2 (7 BHs), 4 (3 BHs), and 6 (2 BHs). SENSE and C-SENSE reconstructions were done inline using the Philips reconstruction software (software release 5.6.1). Imaging parameters are presented in Table 1.

**Table 1:** Scanning parameters

	SENSE-2 16-channel	SENSE-2 72-channel	SENSE-4 72-channel	SENSE-6 72-channel	C-SENSE-2 72-channel	C-SENSE-4 72-channel	C-SENSE-6 72-channel
Field of view (mm)	350 × 350	350 × 350	350 × 350	350 × 350	350 × 350	350 × 350	350 × 350
Reconstructed plane resolution (mm)	0.99	0.99	0.99	0.99	0.99	0.99 – 1.98	0.99 – 1.98
Slice thickness/gap (mm)	8/0.5	8/0.5	8/0.5	8/0.5	8/0.5	8/0.5	8/0.5
Echo time/repetition time (ms)	1.5/2.9	1.5/3.0	1.4/2.8	1.6/3.2	1.5/3.0	1.5/3.1	1.6/3.2
Temporal resolution (ms)	30	30	30	30	30	30	30
Flip angle (degree)	45	45	45	45	45	45	45
Pixel bandwidth (Hz/pixel)	1,894	1,894	2,289	1,701	1,894	1,812	1,603
Cardiac gating	Retrospective	Retrospective	Retrospective	Retrospective	Retrospective	Retrospective	Retrospective
Reconstructed cardiac phases	30	30	30	30	30	30	30
Trajectory	Cartesian	Cartesian	Cartesian	Cartesian	Cartesian	Cartesian	Cartesian
SENSE acceleration factor	2	2	4	6	-	-	-
C-SENSE acceleration factor	-	-	-	-	2	4	6
Receive array coil (+12 posterior)	16 channels	72 channels	72 channels	72 channels	72 channels	72 channels	72 channels
Number of slices	14	14	14	14	14	14	14
Number of breath-holds	7	7	3	2	7	3	2
Total breath-hold time^a^ (s)	70	70	42	28	70	42	28
Slices per breath-hold	2	2	5–4	7	2	5–4	7

^a^Based on heart frequency of 60 beats/min*. C-SENSE* Compressed sensitivity encoding, *SENSE* Sensitivity encoding

### Image analysis

Volumetric quantifications were done offline using Medis Suite 3.1 (Medis Medical Imaging Systems, Leiden, The Netherlands) on a separate post-processing workstation. A sigmoid curve was fitted to the signal intensity at the mid-ventricular LV blood-pool/septum interface during diastole to quantify image sharpness (width of the sigmoid curve, 5–95% intensity range) and blood-to-myocardium contrast normalised to the signal intensity of the myocardium (blood-myocardium)/myocardium, BMC). Visual expert scoring of all slices, graded on a scale of 1 to 5 (1 = nondiagnostic; 2 = suboptimal; 3 = adequate; 4 = good; and 5 = excellent) was independently performed by a radiologist and a cardiologist with focus on endocardial edge definition (EED) and presence of artifacts. Quantitative analysis was performed randomly by a single reviewer, supervised by an experienced radiologist and a cardiologist who specialised in cardiac imaging (experience of both > 15 years). Endo- and epicardial contours were drawn manually on the stack of short-axis slices in end-diastole and end-systole. Segmentation was performed as previously described [24]. LV and RV trabeculations and papillary muscles were included in the blood pools. We analysed the following cardiac metrics: LV and RV end-diastolic volume (EDV), end-systolic volume (ESV), stroke volume (SV), ejection fraction (EF), cardiac output (CO), and LV mass. Assessment of the contour drawing intraobserver variability is provided as Supplemental material.

### Statistical analysis

Data are presented as means and standard deviations or medians and interquartile ranges, as appropriate. The scans acquired with the vendor-provided 16-channel receive array coil and SENSE-2 were considered the reference dataset for pairwise comparisons. Two-sided paired T-tests were used to compare LV and RV EDV, ESV, SV, EF, CO, LV mass, and BMC between methods. Wilcoxon signed-rank tests were used to compare sigmoid width and the image quality scores of EED and the presence of artifacts between methods. Comparisons of BMC and sigmoid width between methods were visualised using Box-and-Whisker Plots. The average range of image quality scores of all slices per subject was visualised using stacked bar charts. To determine agreements between methods, Bland-Altman analyses were used including 95% limits of agreement (LoA) determined by 1.96 × standard deviation of the mean difference and the confidence of the mean difference, determined by the standard error × *t*-value for number degrees of freedom [25–27]. All statistical analyses were performed using R (version 4.0.5) and Rstudio (version 1.3.959). Values of *p* < 0.05 were considered significant for all inference testing and 95% confidence intervals were calculated; *p*-values were adjusted for multiple comparisons using the false discovery rate method as proposed by Benjamini and Hochberg [28].

## Results

**Fig. 2:**
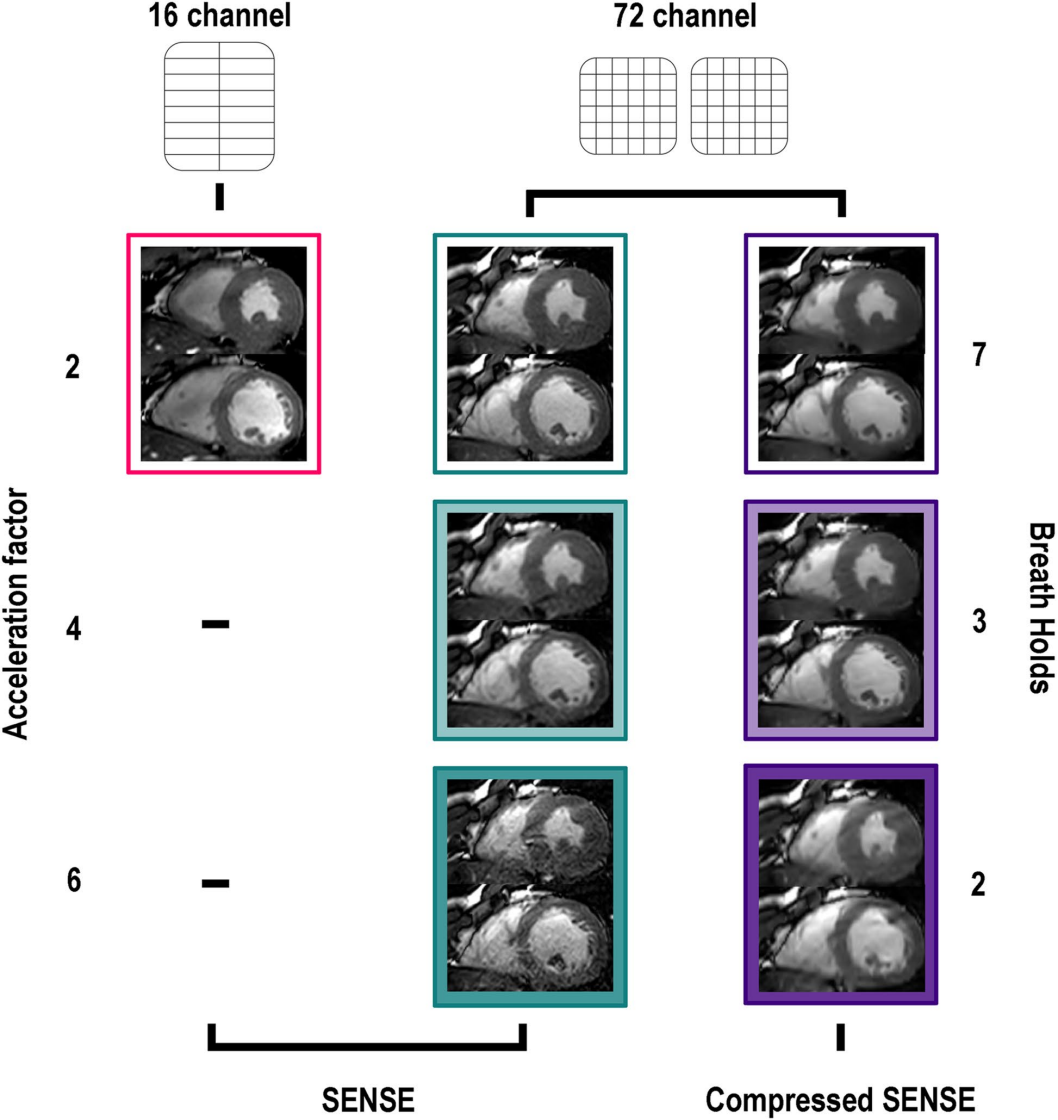
Mid-ventricular balanced steady-state free precession short-axial images of a male participant during end-systole and end-diastole. The reference SENSE-2 scan acquired with the vendor-supplied 16-channel receiver array is outlined in red. Scans acquired with the new 72-channel receiver array are outlined in green for SENSE and purple for C-SENSE

All healthy subjects completed all scans during this study without any technical failures. Subject characteristics can be found in Table 2. A total of 14 short-axis slices covering the whole heart were acquired per subject in 7 BHs for C-SENSE-2, 3 BHs for C-SENSE-4, and 2 BHs for C-SENSE-6. Total BH-time based on an average heart rate of 60 beats/min for C-SENSE-2 was 70 s (7 BHs × 10 s), C-SENSE-4 was 42 s (3 BHs × 15–12 s), and C-SENSE-6 was 28 s (2 BHs × 14 s). Examples of mid-ventricular short-axis images acquired with all test setups of a male participant are shown in Fig. 2. Apical (slice 3), mid-ventricular (slice 7), and basal (slice 11) animations during a full cardiac cycle acquired with all test setups are visualised in supplemental figure S1.

**Table 2:** Characteristics of the study population

Number of subjects	10
Percentage women (%)	50
Age (years)	31.8 ± 8.1^a^
Height (cm)	178.7 ± 8.5^a^
Weight (kg)	74.4 ± 9.9^a^
Body mass index	23.2 ± 1.4^a^
Body surface area (m^2^)	1.9 ± 0.2^a^
Heart rate (beats/min)	60.1 ± 7.3^a^

^a^Data are presented as mean ± standard deviation unless differently specified

### Image sharpness and contrast

**Fig. 3:**
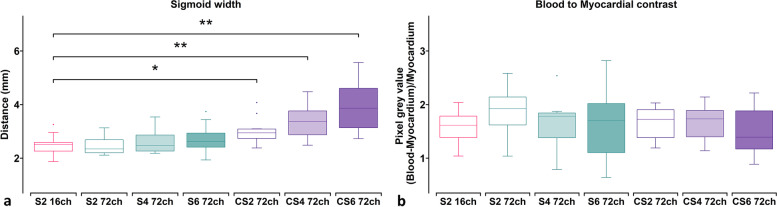
Tukey box plots of (**a**) the width of the sigmoid curves and (**b**) blood-to-myocardium contrast normalised to the myocardium. The vendor-supplied 16-channel receiver array with SENSE-2 was considered the reference group for pairwise comparisons and depicted in red. Scans acquired with the new 72-channel receiver array are depicted in green for SENSE and purple for C-SENSE. *ch* Channel, *CS* Compressed sensitivity encoding, *S* Sensitivity encoding. *p < 0.05, **p < 0.01

Image sharpness assessed by the width of the sigmoid curve was similar (*p* ≥ 0.63 between all SENSE acquisitions (Fig. 3a) and the 16-channel SENSE-2 acquisitions (median 2.5 mm, interquartile range 0.32 mm). The sigmoid width curve was progressively broader and statistically different in C-SENSE-2 (2.95 ± 0.35 mm, *p* < 0.023 and 4 (3.37 ± 0.89 mm, *p* < 0.012) and 6 (3.71 ± 0.95 mm, *p* < 0.001)), compared to the SENSE-2 16-channel receiver array coil. The BMC ratio was essentially the same for all scans compared to the SENSE-2 acquisitions with the 16-channel receive array coil (1.57 ± 0.31, Fig. 3b).

### Image quality

**Fig. 4:**
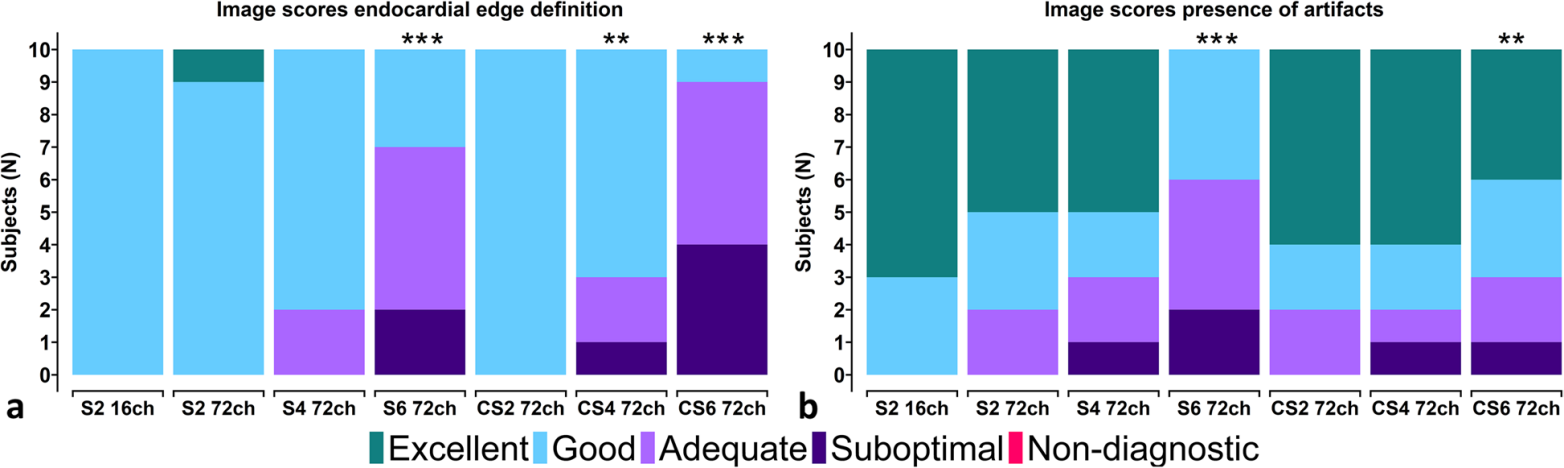
Stacked bar charts of combined averaged image quality scores by the two independent observers. All slices in diastole and systole per subject are averaged where colours depict an image quality score of excellent (green), good (light blue), adequate (light purple), suboptimal (dark purple), or non-diagnostic (red) in each grading criteria based on (**a**) endocardial edge definition and (**b**) presence of artifacts. *p < 0.05, **p < 0.01, ***p < 0.001

Average image quality scores between two experts were similar over all criteria for all scans (*p* = 0.148. Combined averaged scores by both experts in each criterion were used for further analysis. Results for the qualitative imaging scores are shown in Fig. 4a, b. For the reference scan, all data received good to excellent scores for both EED and image artifacts evaluations. These scores were not affected much by the use of the 72-channel receive array coil at an equal two-fold acceleration where only 2 of 10 participants received an image artifact score of 2 (adequate). When going to SENSE-4, 8 of 10 participants received an EED score of 4 (good) and 7/10 participants received an image artifact score of 4 to 5 (good to excellent). For C-SENSE-4, results were somewhat comparable: a small decrease in image quality scores was observed, but still with 7 of 10 participants receiving good to excellent scores for the EED and 8 of 10 participants receiving good to excellent scores for image artifacts. The use of 6-fold acceleration especially further affected EED scores for the C-SENSE case, while for SENSE, most scores (8 of 10) remained adequate or better. Conversely, scores for image artifacts were overall higher for C-SENSE-6, where 7 of 10 participants scored good to excellent compared to SENSE. None of the scans were scored as nondiagnostic on average for both criteria. EED scores were lower in SENSE-6 (median 2.95, effect size *r* 0.873, *p* < 0.001) and C-SENSE 4 (median 3.75, *r* 0.361, *p* < 0.004) and 6 (median 2.2, *r* 0.877, *p* < 0.001) using 72 channels compared to 16 channels and SENSE-2 (median 4). For presence of artifacts, 72-channel median scores were lower in SENSE-6 (median 2.95, *r* 0.873, *p* < 0.001) and C-SENSE-6 (median 2.85, *r* 0.739, *p* < 0.010) channels compared to 16 channels and SENSE-2 (median 4.58).

### Quantitative data

Quantitative data of the LV and RV of all scans are summarised in Tables 3 and 4. All scans with SENSE factors of 2, 4, and 6 acquired with the new 72-channel receive array coil resulted in similar values in LV and RV EF, SV, CO, EDV, ESV, and LV mass compared to SENSE-2 acquisitions with the 16-channel receiver array coil. C-SENSE factors of 2, 4, and 6 also yielded similar values for all cardiac parameters, except for a minor difference in the LV-ESV (-7.9 ± 6.0 mL, *p* < 0.030), the LV-EDV (-6.38 ± 6.0 mL, *p* < 0.047 and the LV-EF (3.2 ± 3.1 %, *p* < 0.047) for C-SENSE-4. Table 4 represents linear regression analyses with R^2^ intercepts and slopes. Table 5 represents the Bland-Altman analyses with the mean bias, the 95% LoA, and confidence of the mean difference of all test setups.

**Table 3:** Quantitative parameters of the left and right ventricle for all test setups

	Test setup						
	SENSE-2 16-channel	SENSE-2 72-channel	SENSE-4 72-channel	SENSE-6 72-channel	C-SENSE-2 72-channel	C-SENSE-4 72-channel	C-SENSE-6 72-channel
LV-EDV (mL)	184.9 ± 23.5	184.8 ± 23.9	183.1 ± 21.4	181.1 ± 23.2	179.1 ± 23.2	178.4 ± 22.5	180.0 ± 15.9
LV-ESV (mL)	73.5 ± 12.9	70.2 ± 15.1	72.1 ± 13.5	70.5 ± 13.9	68.2 ± 13.8	65.0 ± 12.9	64.8 ± 12.1
LV-SV (mL)	111.4 ± 16.5	114.6 ± 13.5	111.0 ± 12.2	110.6 ± 14.8	110.8 ± 16.8	113.4 ± 15.1	115.2 ± 13.1
LV-EF (%)	60.2 ± 4.7	62.3 ± 4.9	60.8 ± 4.3	61.2 ± 4.9	62.0 ± 5.3	63.7 ± 4.7	64.1 ± 5.5
LV-CO (L/min)	6.7 ± 0.9	6.8 ± 0.9	6.7 ± 0.9	6.6 ± 0.9	6.6 ± 1.1	6.8 ± 1.0	6.9 ± 1.2
LV mass (g)	122.9 ± 20.4	122.5 ± 18.1	124.1 ± 20.8	123.1 ± 19.1	124.1 ± 20.4	121.5 ± 20.9	118.5 ± 20.5
RV-EDV (mL)	188.5 ± 18.9	191.5 ± 15.0	192.8 ± 14.5	187.5 ± 14.0	185.4 ± 17.6	184.6 ± 16.3	189.0 ± 14.9
RV-ESV (mL)	77.0 ± 15.0	80.3 ± 11.4	81.2 ± 11.2	80.6 ± 11.8	76.4 ± 13.0	73.4 ± 12.1	79.0 ± 14.4
RV-SV (mL)	110.6 ± 15.4	111.2 ± 13.2	111.7 ± 11.8	106.8 ± 12.2	109.0 ± 14.5	111.1 ± 13.4	110.0 ± 10.6
RV-EF (%)	58.7 ± 6.5	58.1 ± 5.0	57.9 ± 4.6	57.0 ± 5.4	58.8 ± 5.5	60.3 ± 5.3	58.4 ± 5.8
RV-CO (L/min)	6.6 ± 0.8	6.6 ± 1.0	6.7 ± 0.9	6.4 ± 1.0	6.5 ± 1.1	6.7 ± 1.0	6.6 ± 0.9

Data are presented as the mean ± standard deviation. *CO* Cardiac output, *C-SENSE* Compressed sensitivity encoding, *EDV* End-diastolic volume, *EF* Ejection fraction, *ESV* End-systolic volume, *LV* Left ventricle, *RV* Right ventricle, *SENSE* Sensitivity encoding, *SV* Stroke volume

**Table 4:** Linear regression (*y* = *a* + *bx*)

	Reference group: SENSE-2 16-channel																	
	SENSE-2 72-channel			SENSE-4 72-channel			SENSE-6 72-channel			C-SENSE-2 72-channel			C-SENSE-4 72-channel			C-SENSE-6 72-channel		
	*R*^2^	a	b	*R*^2^	a	b	*R*^2^	a	b	*R*^2^	a	b	*R*^2^	a	b	*R*^2^	a	b
EDV/ESV	0.98	0.17	1.00	0.98	3.11	0.99	0.97	3.64	0.96	0.98	-2.48	0.98	0.98	-4.8	0.99	0.97	-0.59	0.99
SV	0.70	34.2	0.71	0.77	38.1	0.66	0.51	40.4	0.62	0.78	14.00	0.87	0.80	22.5	0.81	0.60	46.3	0.59
EF	0.61	15.6	0.75	0.71	17.7	0.70	0.56	15.3	0.74	0.59	18.3	0.72	0.67	15.3	0.78	0.57	7.88	0.89
CO	0.24	2.98	0.56	0.37	2.27	0.66	0.23	2.73	0.57	0.46	0.89	0.86	0.44	1.00	0.87	0.35	1.54	0.79
LV mass	0.93	16.70	0.86	0.91	4.45	0.97	0.93	11.6	0.91	0.91	2.98	0.98	0.88	5.98	0.95	0.95	-1.71	0.98

Data are presented as LV and RV variables combined for EDV/ESV, SV, EF, and CO. *a* intercept, *b* slope, *CO* Cardiac output, *C-SENSE* Compressed sensitivity encoding, *EDV* End-diastolic volume, *EF* Ejection fraction, *ESV* End-systolic volume, *LV* Left ventricle, *RV* Right ventricle, *SENSE* Sensitivity encoding, *SV* Stroke volume

**Table 5:** Pairwise comparison and Bland-Altman analyses of left and right ventricular quantitative parameters

	Reference group: SENSE-2 16-channel																	
	SENSE-2 72-channel			SENSE-4 72-channel			SENSE-6 72-channel			C-SENSE-2 72-channel			C-SENSE-4 72-channel			C-SENSE-6 72-channel		
	Mean bias ± LoA	Conf.	*p*-value	Mean bias ± LoA	Conf.	*p*-value	Mean bias ± LoA	Conf.	*p*-value	Mean bias ± LoA	Conf.	*p*-value	Mean bias ± LoA	Conf.	*p*-value	Mean bias ± LoA	Conf.	*p*-value
LV-EDV (mL)	-0.10 ± 16.11	5.98	0.970	-1.79 ± 17.45	6.48	0.900	-3.86 ± 18.31	6.79	0.827	-6.82 ± 12.71	4.72^×^	0.058	-6.38 ± 11.68	4.33^×^	0.047*	-4.11 ± 10.70	7.68	0.541
LV-ESV (mL)	-3.29 ± 13.14	4.88	0.964	-1.41 ± 10.48	3.89	0.900	-3.01 ± 9.62	3.57	0.827	-7.12 ± 12.23	4.54^×^	0.058	-7.94 ± 11.73	4.35^×^	0.030*	-7.41 ± 13.82	5.13^×^	0.058
LV-SV (mL)	3.19 ± 18.68	6.93	0.964	-0.38 ± 16.83	6.25	0.965	-0.85 ± 19.64	7.29	0.881	0.30 ± 14.28	5.30	0.909	1.56 ± 12.42	4.61	0.840	3.30 ± 22.05	8.18	0.698
LV-EF (%)	2.05 ± 7.82	2.90	0.964	0.58 ± 5.67	2.11	0.900	0.96 ± 6.47	2.40	0.881	2.50 ± 6.20	2.30^×^	0.146	3.16 ± 6.12	2.28^×^	0.047*	3.32 ± 8.21	3.05^×^	0.109
LV-CO (L/min)	0.14 ± 1.94	0.71	0.970	-0.01 ± 1.65	0.61	0.973	-0.07 ± 1.74	0.65	0.881	-0.03 ± 1.58	0.59	0.909	0.14 ± 1.54	0.59	0.840	0.26 ± 2.17	0.80	0.667
LV mass (g)	-0.39 ± 10.63	3.94	0.970	1.24 ± 12.55	4.66	0.900	0.19 ± 10.33	3.83	0.910	0.69 ± 12.08	4.48	0.894	-0.83 ± 14.34	5.32	0.926	-4.21 ± 8.74	3.24^×^	0.698
RV-EDV (mL)	2.97 ± 22.06	8.19	0.964	4.29 ± 19.91	7.39	0.900	-1.09 ± 27.66	10.26	0.827	-3.63 ± 24.75	9.19	0.894	-4.31 ± 19.51	7.24	0.468	1.32 ± 20.52	7.61	0.827
RV-ESV (mL)	2.36 ± 13.80	5.12	0.964	3.23 ± 14.75	5.47	0.900	2.66 ± 15.54	5.77	0.827	-2.76 ± 20.28	7.53	0.894	-4.40 ± 14.88	5.52	0.324	2.10 ± 16.74	6.21	0.698
RV-SV (mL)	0.62 ± 14.73	5.47	0.970	1.06 ± 13.87	5.15	0.900	-3.75 ± 24.39	9.05	0.827	-0.86 ± 15.50	5.75	0.894	0.10 ± 15.06	5.59	0.970	-0.78 ± 16.37	6.07	0.838
RV-EF (%)	-0.66 ± 5.12	1.90	0.964	-0.79 ± 6.03	2.24	0.900	-1.69 ± 8.13	3.02	0.827	0.57 ± 7.78	2.89	0.894	1.40 ± 6.50	2.41	0.216	-0.67 ± 7.00	2.60	0.741
RV-CO (L/min)	-0.01 ± 1.57	0.58	0.970	0.01 ± 1.35	0.50	0.900	-0.22 ± 1.85	0.68	0.881	-0.08 ± 1.47	0.54	0.894	0.08 ± 1.64	0.61	0.926	-0.02 ± 1.15	0.43	0.888

Data are presented as the mean bias, 95% LoA of the mean bias, and standard error × *t* as confidence of the mean difference. *CO* Cardiac output, *Conf.* Confidence of the mean difference *C-SENSE* Compressed sensitivity encoding, *EDV* End-diastolic volume, *EF* Ejection fraction, *ESV* End-systolic volume, *LoA* Limits of agreement, *LV* Left ventricle, *RV* Right ventricle, *SENSE* Sensiitivity encoding, *SV* Stroke volume. *p < 0.05, ^×^systematic bias: line of equality (0) outside the confidence intervals of the mean difference

**Fig. 5:**
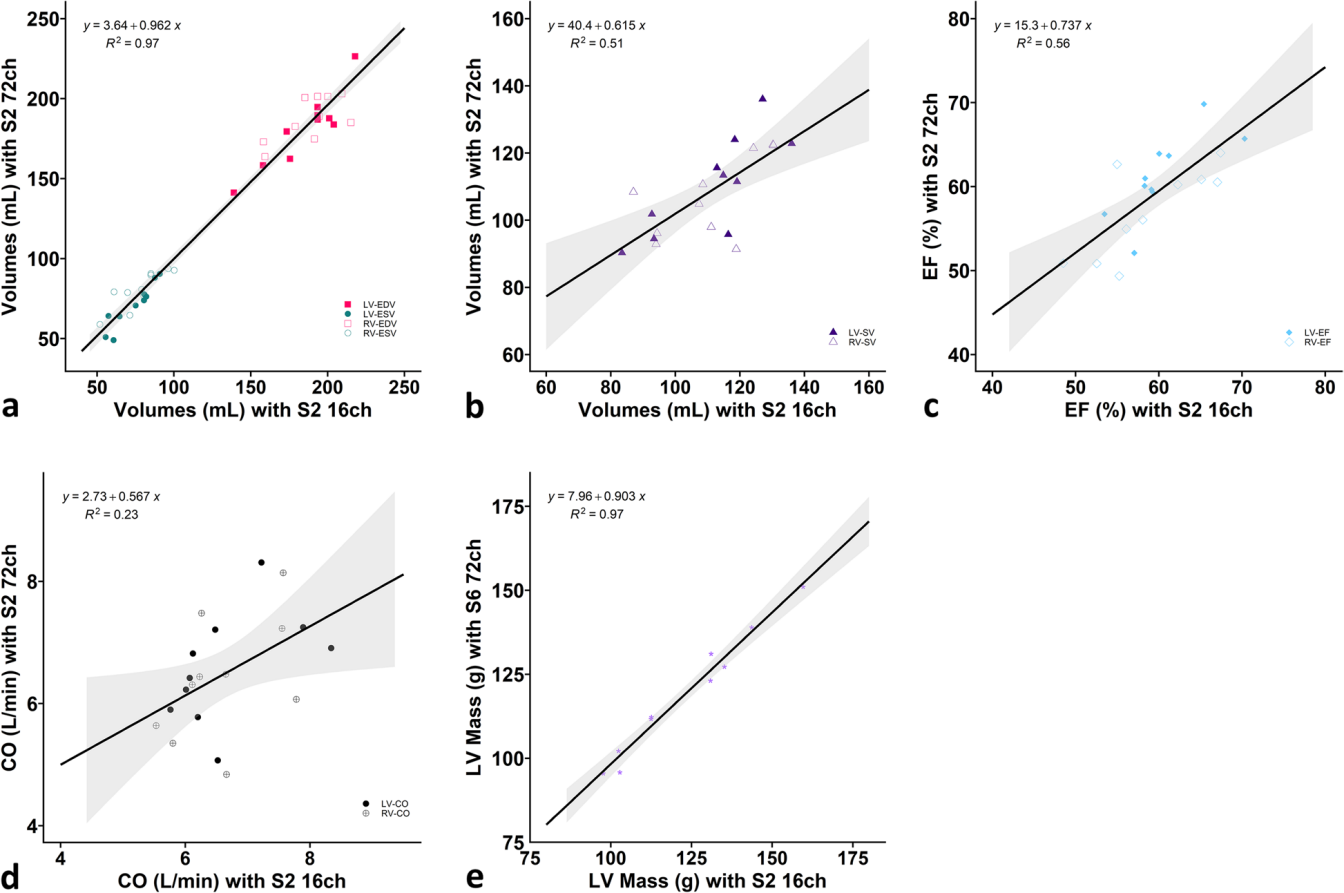
Linear regression plots including 95% confidence intervals comparing (**a**) left ventricular (LV) and right ventricular (RV) end-diastolic volumes (EDV) and end-systolic volumes (ESV), (**b**) LV and RV stroke volumes (SV), (**c**) LV and RV ejection fractions (EF), (**d**) LV and RV cardiac outputs (CO), and (**e**) LV mass measured with SENSE-2 16-channel receiver array (7 breath-holds) as reference compared to SENSE-6 72-channel receiver array (2 breath-holds). *ch* Channel, *CS* Compressed sensitivity encoding, *S* Sensitivity encoding

**Fig. 6:**
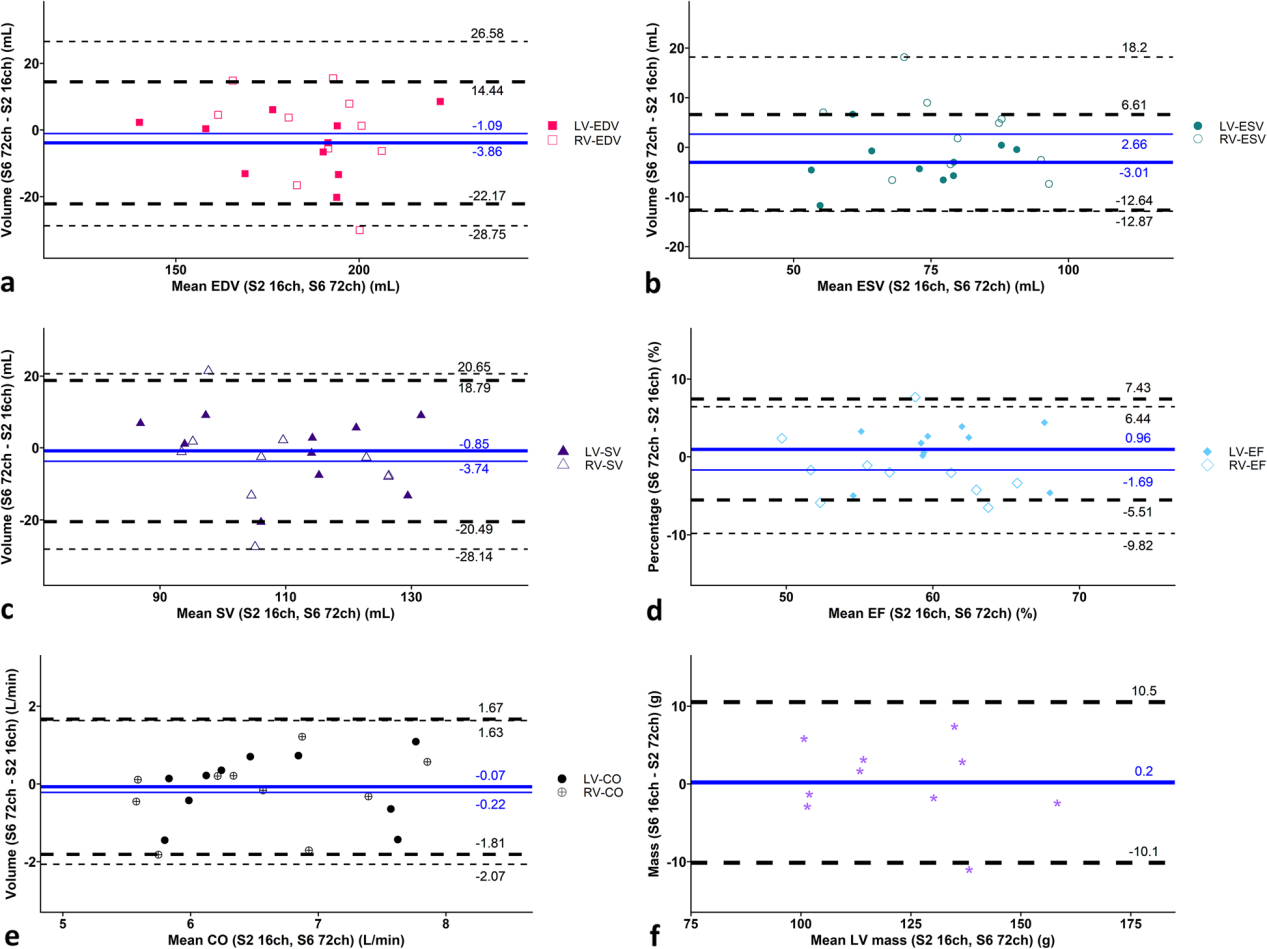
Bland-Altman plots comparing (**a**) left ventricular (LV) and right ventricular (RV) end-systolic volumes (ESV), (**b**) LV and RV end-diastolic volumes (EDV), (**c**) LV and RV stroke volumes (SV), (**d**) LV and RV ejection fractions (EF), (**e**) LV and RV cardiac outputs (CO), and (**f**) LV mass measured with SENSE-2 16-channel receiver array (7 breath-holds) as reference compared to SENSE-6 72-channel receiver array (2 breath-holds). The solid blue line indicates the mean bias (thick = LV, thin = RV), with the dashed black lines showing the upper and lower limits of agreement, determined as mean bias ± 1.96 × standard deviation, between the two techniques (thick dashed = LV, thin dashed = RV). Confidence intervals of the mean difference in the Bland-Altman plots are presented in Table 5. *ch* Channel, *CS* Compressed sensitivity encoding, *S* Sensitivity encoding

Linear regression and Bland-Altman analyses of the LV and RV ESV, EDV, SV, EF, and LV mass of SENSE-6 acquired with the 72-channel receive array coil are visualised in Figs. 5a–d and 6a–f. For SENSE-6 and 72 channels correlations varied from excellent (*R*^2^ 0.97 for LV-ESV and RV-ESV as well as LV-EDV and RV-EDV; *R*^2^ 0.93 for LV mass) to medium (*R*^2^ 0.56 for LV-EF and RV-EF; *R*^2^ 0.51 for LV-SV and RV-SV; *R*^2^ 0.23 for LV-CO and RV-CO). LV-EDV and RV-EDV were underestimated by -3.86 ± 18.31 mL and -1.09 ± 18.31, respectively, whereas the LVESV- and RV-ESV differed by -3.01 ± 9.62 mL and 2.66 ± 15.54 mL, LV-SV and RV-SV by -0.85 ± 19.64 and -3.75 ± 24.39 mL, LV-EF and RV-EF by 0.96 ± 6.47 % and -1.69 ± 8.13 %, LV-CO and RV-CO by -0.07 ± 1.74 L/min and -0.22 ± 1.85 L/min, the LV mass by 0.19 ± 10.33 g, respectively. No systematic bias was present when comparing the SENSE-6 channel scans with the SENSE-2 16-channel scans.

## Discussion

In this prospective study, we demonstrated diagnostic image quality with accurate determination of biventricular function and morphology in just 2 BHs using a newly developed 72-channel receiver array coil. Biventricular volumetric indices using a cine bSSFP acquisition with SENSE-4 (3 BHs) and 6 (2 BHs) were similar to the reference acquired with SENSE-2. A minor underestimation of the LV-EDV and/or LV-ESV with C-SENSE-2 (7 BHs), C-SENSE-4 (3 BHs), and 6 (2 BHs) was found as seen in the mean biases and confidence of the Bland-Altman analyses. As a result, the LV-EF was slightly overestimated with C-SENSE-2, 4, and 6. LV mass was only slightly underestimated with C-SENSE-6. Taken together, the C-SENSE accelerated sequences were adequate for the quantification of LV, SV, and CO as well as all RV volumetric indices.

Usability of a 2-BH approach with (C-)SENSE-6 was confirmed by the Bland-Altman analyses. Our findings can be compared to other CMR cine studies that investigated acquisition acceleration. Several studies comparing prototype C-SENSE bSSFP sequences with acceleration factors up to 8, reported mean biases in LV indices ranging from 0.23 to 14.77 mL for the LV-EDV, 0.39–8.38 mL for the LV-ESV, 0.62–8.70 mL for the LV-SV, 0.36–2.0% for the LV-EF, and 0.51–8.0 g for the LV mass [12, 14, 16–18]. Our protocols resulted in similar small bias values, which will have little to no clinical impact. Also, the 95% LoAs of the intraobserver variability in contour drawing were comparable to previously shown values in an identical healthy population (age 29.9 ± 4.5, mean ± standard deviation; *n* = 10) [29]. In the latter study, a similar, not accelerated clinically-used CMR protocol was scanned twice by the same operator. The 95% LoA were 12.05 mL for LV-EDV, 8.93 mL for LV-ESV, 4.54% for LV-EF, and 14.09 g for LV mass, which values primarily resulted from slice-planning variations rather than from the contour drawing. These values are in line with our intraobserver variability in contour drawing (Supplementary Table S2), albeit with lower 95% LoA values. For some of the parameters, the 95% LoA values increased when acceleration increased to a factor of 6. Yet, even these LoA values were still comparable to other studies [12, 14, 16, 17], which reported LoA values ranges of 3.76–16.3 mL for LV-EDV, 4.08–11.33 mL for LV-ESV, 3.04–10.71 mL for LV-SV, 1.25–3.06 % for LV-EF, and 4.28–11.82 g for LV mass. Thus, 6 times acceleration has some impact on accuracy which was also reflected by a lower image sharpness (lower EED values), resulting in less well-defined blood-myocardial borders. However, in the current study, we also repeated the slice planning after switching coils. Thus, the increase in variation could also be partly due to replanning of the imaging planes as discussed previously [29]. Importantly, our results show that there is no systematic bias and 95% LoA values are clinically acceptable for acquisitions up to SENSE-6.

We aimed at keeping the BHs relatively short (< 15 s) since long BHs are not well tolerated by a large group of patients, although we could have condensed the acquisition into one long BH as is done in some other approaches [30]. Higher accelerations have been achieved using prototype C-SENSE bSSFP sequences and other experimental accelerated two- and three-dimensional sequences that usually require dedicated offline non-vendor-supported reconstruction software [14, 16–18, 30–32]. Often the number of BHs is reduced at the expense of very long BH times and decreased temporal resolution [30–32]. Note that the implementation of C-SENSE used in this study is a frame-by-frame reconstruction and does not exploit sparsity in the temporal domain.

For scans with the highest accelerations, the typical SENSE unfolding artifacts became apparent (Fig. 2 and supplementary Fig. S1), particularly for the SENSE accelerated scans, even with the use of the 72 (+12) receive array coil. Nevertheless, an acceleration factor of 6 is high for a two-dimensional Cartesian k-space acquisition, and therefore, it is to be expected that the reconstruction starts to break down when less than 20% of the original k-space is sampled [23, 33]. Higher acceleration factors may be achieved by including undersampling and sparsity in the temporal domain or by three-dimensional acquisition and reconstruction, but these are currently not standard available and would require (lengthy) offline reconstructions. Despite some artifacts arising with higher acceleration factors, the cine images were scored diagnostically adequate by the two independent expert readers. We therefore think that the present implementation using moderate to high acceleration factors (*e.g.,* C-SENSE-4, SENSE-6) provides a good balance between diagnostic relevance and required scan time to determine biventricular morphology and function quantification.

Because the sequence parameters (repetition time, echo time, flip angle) were nearly identical for the different scans, the BMC was preserved for the accelerated scans, which facilitated delineation of endocardial and epicardial borders in all cases. Our accelerated CMR cine acquisition therefore does not require contrast injection for the quantification of LV and RV functional parameters like recently introduced three-dimensional accelerated scans [31]. Image sharpness—assessed by fitting a sigmoid curve to the image intensity at the septum-blood pool border—was preserved for the SENSE-accelerated scans, but as expected, decreased with increasing C-SENSE acceleration due to smoothing from spatial undersampling and compressed-sensing reconstruction. This was reflected by the EED scores that were lower for the C-SENSE accelerated scans. Additionally, because k-space filling at high accelerations becomes very low, the C-SENSE reconstruction algorithm introduced some variation in the reconstructed pixel sizes between roughly 1 and 2 mm (see Table 1). Together this resulted in scores that were lower for the C-SENSE accelerated scans.

It has been shown that a higher channel count can increase the signal-to-noise ratio and decrease the g-factor facilitating higher scan acceleration [19, 21, 22]. However, current clinical CMR studies use vendor-supplied receive array coils typically up to a maximum of 32 channels. Our newly developed 72-channel receive array coil is designed to interface with every clinical system. This will give any hospital the possibility to upgrade their current system to allow accelerated state-of-the-art CMR without the need to replace their current scanners.

This study is the first step towards implementing a dedicated high channel number receive array coil and high scan acceleration in a routine clinical CMR workflow. We will focus future studies on further acceleration of additional clinical scans, including T1 mapping and T2 mapping. Additionally, we will use the acceleration to acquire free-breathing bSSFP cine images with a sufficient spatial resolution to quantify biventricular morphology and function with real-time frame rates and further exploit the high receive channel number to accelerate three-dimensional acquisitions [33].

There are some limitations to our study. First, the study population consisted of a small sample size and included only young healthy volunteers with normal body mass index, without any known cardiovascular disease. In the future, a larger cohort of patients with cardiovascular diseases should be included, involving subjects with arrhythmias, wall motion abnormalities, defective heart valves, and impaired BH capacity [34]. Arrhythmias could induce electrocardiographic mistriggering causing jumps in repetion time or heart-rate cycle variations which result in inconsistencies between k-space segments leading to artifacts. Blood flow impairments caused by wall motion abnormalities and/or defective heart valves may introduce intra-voxel phase dispersion causing signal loss artifacts. Also, impaired respiratory capacity can be problematic specifically during multiple slice acquisitions using high acceleration factors with SENSE, where discrepancies between the calibration scan and the image acquisition may occur. Second, the C-SENSE scanning with increasing acceleration factors was not performed randomly, potentially introducing a scan-order bias. Finally, all scans were performed by the same operator, and variability from variation in short-axis planning was not investigated.

Taken together, our findings show that quantification of biventricular morphology and function is clinically feasible for diagnostic purposes in just 2 BHs with a 72-channel receive array coil and the vendor-supplied C-SENSE-6 acceleration, where SENSE slightly outperformed C-SENSE.

## Supplementary Information

**Additional file 1: Figure S1.** Full cardiac cycle animations at apical (slice 3), mid-ventricular (slice 7) and basal (slice 11) short-axis field of views in a male participant. **Table S1.** Repeated measure comparison of left and right ventricular quantitative variables. **Table S2.** Linear regression intra-observer contour tracing SENSE=2 16-channel array cine data (Y = a + bx).

## Data Availability

Analysed datasets can be made available by the corresponding author on plausible request.

## Abbreviations

BH: Breath hold
BMC: Blood-to-myocardium contrast
bSSFP: Balanced steady-state free precession
CO: Cardiac output
C-SENSE: Compressed sensitivity encoding
EDV: End-diastolic volume
EED: Endocardial edge definition
EF: Ejection fraction
ESV: End-systolic volume
LoA: Limits of agreement
LV: Left ventricle
RV: Right ventricle
SENSE: Sensitivity encoding
SV: Stroke volume
